# Supplementary material for: Low First Trimester Pregnancy-Associated Plasma Protein-A Levels Are Not Associated with an Increased Risk of Intrapartum Fetal Compromise or Adverse Neonatal Outcomes: A Retrospective Cohort Study
Source: J Clin Med. 2020 Apr 13;9(4):1108. doi: 10.3390/jcm9041108 (PMC7230680; doi:10.3390/jcm9041108)
Supplement: Supplementary file 1 [file jcm-09-01108-s001.pdf]

Supplementary Table S1. Intrapartum and perinatal outcomes for women with SGA infants (BW < 10th centile).

	**PAPP-A ≤ 0.4 MoM**	**PAPP-A > 0.4 MoM**	*p*	**Adjusted OR (95% CI)**	*p*
n	69	800			
**Mode of birth**					
Spontaneous vaginal*	39.1 (27)	50.3 (402)	0.08	0.65 (0.39–1.08)	0.10
Instrumental*	21.7 (15)	21.3 (170)	0.92	1.05 (0.58–1.91)	0.88
All Vaginal Births*	60.9 (42)	71.5 (572)	0.06	0.64 (0.38–1.08)	0.10
Planned CS*	15.9 (11)	8.4 (67)	0.04	1.98 (0.98–3.97)	0.06
Emergency CS*	23.2 (16)	20.1 (161)	0.54	1.16 (0.64–2.11)	0.62
*Emergency CS IFC**	1.5 (1)	1.9 (15)	1.00	0.80 (0.10–6.20)	0.83
**Gestation at birth (weeks)**	38 (37–39)	39 (38–40)	<0.001	-	-
Preterm birth*	17.4 (12)	5.8 (46)	<0.001	3.25 (1.57–6.70)	0.001
**Birthweight**	2405.8 (481.0)	2661.6 (376.5)	<0.001	-	-
Birthweight <5th centile*	62.3 (43)	44.3 (354)	0.004	2.14 (1.29–3.54)	0.003
**SCNO^**	5.8 (4)	7.8 (62)	0.56	0.41 (0.11–1.51)	0.18

Data presented as %(*n*), Odds ratio (95% Confidence interval); PAPP-A MoM; Pregnancy-associated plasma protein-A multiples of the median; OR, odds ratio; CI confidence interval; CS, caesarean section; IFC, intrapartum fetal compromise; SCNO=acidosis, NICU admission, 5-minute Apgar<3, perinatal death * adjusted for hypertension. ^ adjusted for hypertension, gestation and birthweight.
